# The Molecular Mechanism Underlying Continuous Exercise Training-Induced Adaptive Changes of Lipolysis in White Adipose Cells

**DOI:** 10.1155/2015/473430

**Published:** 2015-05-13

**Authors:** Junetsu Ogasawara, Tetsuya Izawa, Tomonobu Sakurai, Takuya Sakurai, Ken Shirato, Yoshinaga Ishibashi, Hitoshi Ishida, Hideki Ohno, Takako Kizaki

**Affiliations:** ^1^Department of Molecular Predictive Medicine and Sport Science, Kyorin University, School of Medicine, Mitaka, Tokyo 181-8611, Japan; ^2^Graduate School of Health and Sports Science, Doshisha University, Kyotanabe, Kyoto 610-0394, Japan; ^3^Faculty of Culture and Sport Policy, Toin University of Yokohama, Yokohama, Kanagawa 225-8503, Japan; ^4^Department of Third Internal Medicine, Kyorin University, School of Medicine, Mitaka, Tokyo 181-8611, Japan

## Abstract

Physical exercise accelerates the mobilization of free fatty acids from white adipocytes to provide fuel for energy. This happens in several tissues and helps to regulate a whole-body state of metabolism. Under these conditions, the hydrolysis of triacylglycerol (TG) that is found in white adipocytes is known to be augmented via the activation of these lipolytic events, which is referred to as the “lipolytic cascade.” Indeed, evidence has shown that the lipolytic responses in white adipocytes are upregulated by continuous exercise training (ET) through the adaptive changes in molecules that constitute the lipolytic cascade. During the past few decades, many lipolysis-related molecules have been identified. Of note, the discovery of a new lipase, known as adipose triglyceride lipase, has redefined the existing concepts of the hormone-sensitive lipase-dependent hydrolysis of TG in white adipocytes. This review outlines the alterations in the lipolytic molecules of white adipocytes that result from ET, which includes the molecular regulation of TG lipases through the lipolytic cascade.

## 1. Introduction

Obesity, which results from the energy intake that is in excess of energy expenditure, is a major global health problem not only in developed nations (western world) but in low- and middle-income countries (less developed countries) [1]. White adipocytes are capable of storing excess energy as triacylglycerol (TG), and they play a key role in energy metabolism by providing free fatty acids (FFA) and glycerol through the hydrolysis of TG. The induction of lipolysis, as well as the inhibition of TG synthesis in white adipocytes, has been considered a target of therapy for the prevention and improvement of obesity and its related disorders. Therefore, clarifying the mechanisms underlying the physical exercise-induced alteration of lipolytic molecules in white adipocytes would be useful for establishing a new method for exercise therapy as well as for understanding the biological meanings of the lipolytic events themselves.

This elucidation of lipolysis has demonstrated how ectopic lipid accumulation in skeletal muscle and liver is closely associated with insulin resistance syndrome and diabetes [2]. In particular, the flux of muscular fatty acids is known to play a pivotal role in the development of the abnormalities of muscle and whole-body energy metabolism [3], demonstrating that an increase in the consumption of intramuscular lipids via mitochondrial *β*-oxidation would be beneficial for the prevention of obesity-related disorders. Indeed, in previous studies, ET has been shown to increase the metabolic utilization of lipids in both healthy [4] and obese humans [5] and reduce lipid storage in liver [6]. In addition, it appears that ET-induced loss of the absolute content of lipid in white adipocytes per se has a positive effect on reducing the levels of the redistribution of lipids in other tissues through an attenuation of synthetic substrate content, that is, FFA and glycerol. Thus, ET would be a highly effective tool for reducing ectopic fat accumulation and/or increasing the hydrolysis of TG in white adipocytes themselves.

The molecular mechanisms underlying lipolysis in white adipocytes are known to be regulated mainly by hierarchical activation of the lipolytic cascade, which is modified through both an *α*- and *β*-AR-cAMP production system, thereby distally exerting a changeover to the hydrolytic action of lipases. The stimulation of these two ARs induces opposite effects: *α*-antilipolytic and *β*-lipolytic (details are described in the next section). Of note, it has been shown that complete activation of lipolysis in white adipocytes is only obtained when catecholamines were in the presence of an *α*_2_-AR antagonist in human, although there are no changes observed in rodents [7]. This *α*_2_-antilipolytic component counteracting the *β*-AR-mediated lipolysis has been well known as the “*α*_2_/*β*-adrenergic balance” [8]. On the other hand, Arner and colleagues [9] have shown that *α*_2_-ARs modulate lipolysis at rest, whereas the *β*-ARs modulate lipolysis during physical exercise, even if *α*_2_/*β*-adrenergic balance exists in human white adipocytes. In addition, it has been demonstrated that the number of *α*_2_-ARs in jerboa, dormouse, and rat is lower than that in humans [10], suggesting that catecholamines-induced lipolysis in rodents, which have low number of *α*_2_-ARs, is regulated through alteration in *β*-lipolytic function. Therefore, the experiments using the jerboa, dormouse, and rat would be helpful for elucidation of physical exercise-induced molecular changes in lipolytic molecules in human white adipocytes, because *β*-ARs modulate lipolytic response during physical exercise in humans [9]. It has been widely accepted that ET facilitates hormone-stimulated lipolysis in white adipocytes in mammals [11, 12]. However, the effects of ET on both the molecular behavior and on the expression levels of lipolytic molecules in white adipocytes remain to be missing pieces of the puzzle, although recent evidence has identified both new lipase and lipolytic cofactors: adipose triglyceride lipase (ATGL) [13], PAT family proteins [14], comparative gene identification-58 (CGI-58) [15], and lipotransin [16].

The purpose of this review is to marshal the fact that exercise training (ET) induced changes in the lipolytic molecules via *β*-AR, commonly expressed in white adipocytes of human and rodents. The introduced results of this review are mixed with those obtained from human and experimental animals. However, the consideration of this viewpoint appears to enable a deepening of understanding lipolytic events in white fat cells by ET, because ET-induced adaptive changes of lipolytic molecules in white adipocytes are a universal mechanism in mammalian species. Together, first, the currently known mechanism(s) of the lipolytic cascade and the molecular behavior of lipases and cofactors are outlined. Then, attention is focused on the ET-induced adaptive changes of lipolytic molecules, which were mainly obtained from our studies of white adipocytes.

## 2. Basic Structure of the Lipolytic Cascade in White Adipocytes

**Figure 1:**
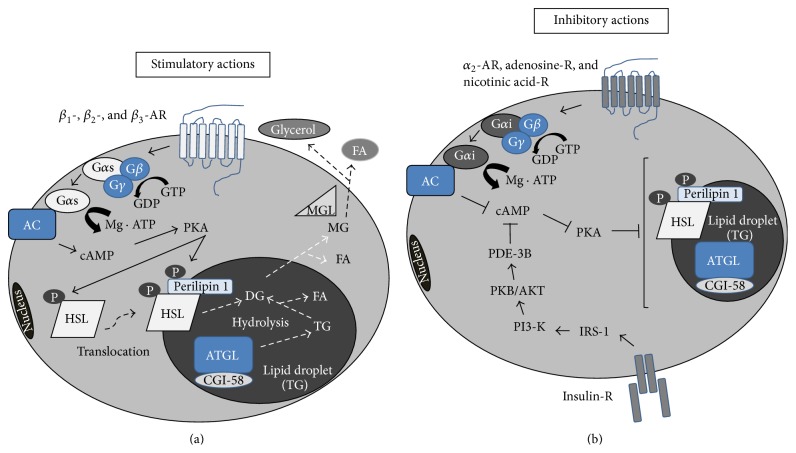
Lipolysis in white adipocytes is mainly regulated through GPCRs that localize on the plasma membrane. (a) Under stimulatory conditions, ligands binding to GPCRs, that is, *β*_1_-, *β*_2_-, and *β*_3_-AR, activate AC through the action of G*α*s, resulting in an increase in PKA activity through the accumulation of intracellular cAMP, and, in turn, PKA phosphorylates and activates HSL. Phosphorylated HSL translocates on the lipid droplet and thereby activates lipolysis. (b) On the other hand, ligands binding to GPCRs, that is, *α*_2_-AR, adenosine-R, and nicotinic acid-R, attenuate lipolysis via a reduction in cAMP production. Insulin receptor signaling also inhibits lipolytic response via the activation of PDH-3B, a cAMP-degrading enzyme.

Lipolysis in white adipocytes is regulated by a multifaceted phenomenon that is subject primarily to distinct temporal controls such as hormonal stimulation via catecholamines. The hormonal activation of lipolysis in adipocytes is mediated via a traditional cAMP-dependent signal transduction process [17, 18] (Figure 1(a)). The stimulation of G-protein coupled receptors (GPCRs), that is, *β*_1_-, *β*_2_-, and *β*_3_-adrenergic receptors (*β*-ARs), induces a conformational change in the G*α* subunit of the heterotrimeric G protein (G*αβγ*) that leads to GDP release and GTP binding. Activated G*α*s leads to the activation of adenylyl cyclase (AC) and to the production of cAMP. However, the stimulation of GPCRs, that is, *α*_2_-adrenergic receptor [19], adenosine receptor [20], and prostaglandin E2 receptor [21], which stimulate G*α*i, causes the inactivation of AC and reduces the production of cAMP, resulting in an attenuation of the lipolytic response. In addition, insulin attenuates intracellular cAMP production through increases in phosphodiesterase-3B (PDE-3B) activity, which changes cAMP to AMP via the activation of protein kinase B/AKT (Figure 1(b)). An increased intracellular cAMP level phosphorylates and activates cAMP-dependent protein kinase A (PKA) [22, 23] and subsequently phosphorylates hormone-sensitive lipase (HSL); it is well known that the phosphorylation of HSL at Ser563, Ser659, and Ser660, by cAMP-dependent protein kinase (PKA), enhances its enzymatic activity and that extracellular-regulated kinase (ERK) induces the phosphorylation of HSL at Ser600 in 3T3-L1 adipocytes [24], although there are no studies supporting this result in primary mammalian white adipocytes. Phosphorylated HSL activates the hydrolysis of TG in adipocytes [25] through the translocation of HSL from the cytoplasm to the surface of lipid droplets [26]. On the other hand, an inhibitory effect of insulin has been reported on HSL activity [27, 28], and AMP activated protein kinase (AMPK) attenuates HSL activity through an increase in its phosphorylation at Ser565 [29].

**Figure 2:**
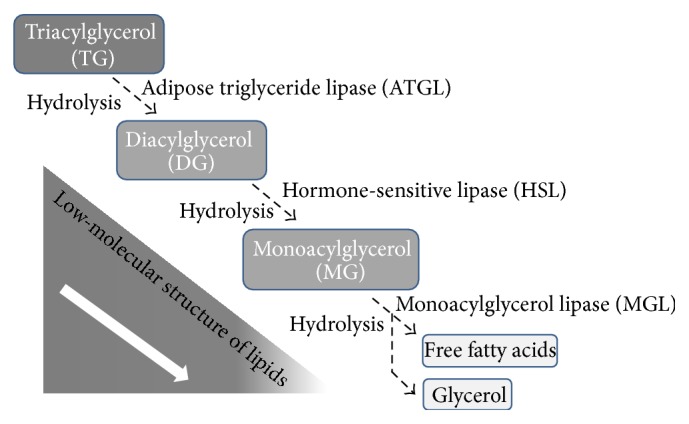
ATGL acts exclusively on the hydrolysis of TG. A major component of HSL activity depends on the generation of DG, a substrate from the action of ATGL. Finally, MGL acts to liberate glycerol and the final FFA.

In 2004, three groups independently published the discovery of an enzyme that could hydrolyze TG [13, 30, 31] and named it adipose triglyceride lipase (ATGL). Unlike HSL, ATGL has no specificity for the hydrolysis of MG, cholesterol esters, or retinyl esters. ATGL does, however, have a substrate specificity for TG that is 10-fold higher than that for DG [3], indicating that it selectively acts as the first step in TG hydrolysis and that its hydrolytic function is not restricted to the catabolism of lipid droplets [32] in adipose tissue. Moreover, two phosphorylation sites of ATGL, at Ser404 and Ser428, have been identified in the C-terminal region in humans [13, 33]. In contrast with HSL, however, the functional roles of enzyme phosphorylation, as it involves protein kinases, remain unknown. Together, both HSL and ATGL act hierarchically to regulate TG hydrolysis: ATGL initiates lipolysis by removing the first FA from TG to, in turn, produce DG; HSL generates an additional FA from DG and MG to produce glycerol (Figure 2). In these events, the phosphorylation of lipases plays a central role in the regulation of enzyme activity and is closely associated with the catabolism of adipocytes.

## 3. Regulation of Lipolysis via the Coordinated Action of Lipases and Cofactors

**Figure 3:**
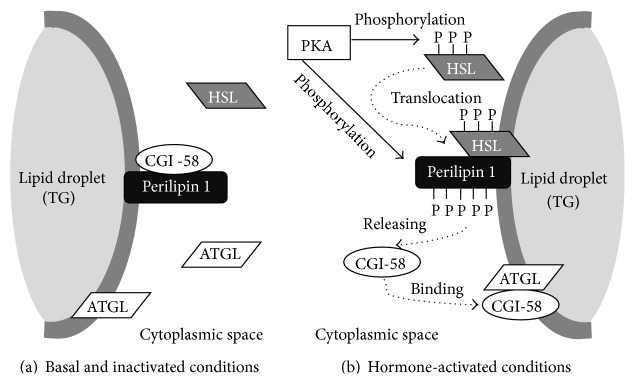
Under basal and inactivated conditions, perilipin 1 and CGI-58 form a complex on the surface of lipid droplets (a). On the other hand, PKA activation leads to the phosphorylation of both HSL and perilipin 1, resulting in HSL and perilipin 1 forming a complex on the surface of lipid droplets. Released CGI-58 from phosphorylated perilipin 1 binds to ATGL to induce lipolysis (b).

The discovery of perilipin 1 provided proof of cofactors which exist in the cytoplasm and on the lipid droplet surface [34]. Perilipin 1 is the founding member of the perilipin, adipophilin, and TIP47 family (referred to as the PAT/perilipin family protein) of lipid droplet-coated proteins [35] and is expressed mostly in white adipose tissue, where it coats lipid droplets, and in steroidogenic tissue [36]. Perilipin 1 has as many as six phosphorylation sites (Ser81, Ser222, Ser276, Ser433, Ser492, and Ser517) in adipocytes by PKA [37–40]. Several studies have reported that perilipin 1 is multifunctional and is capable of reducing basal lipolysis via combining HSL with lipid droplets to form a barrier [41] and promotes the lipolysis movement of perilipin 1 away from fat droplets [42] through lipase-dependent and -independent mechanisms [43]. Moreover, the CGI-58, also known as *α*/*β* hydrolase domain-containing protein 5 (ABHD5), was found to increase the TG hydrolase activity of ATGL owing to a direct interaction with ATGL proteins [44]. CGI-58 also has the ability to be associated with perilipin 1 [35, 45–47], demonstrating that the localizations of both perilipin 1 and CGI-58 are centrally involved in the organization and regulation of lipolytic effector interactions in both basal and hormone-stimulated states. The conceptual consensus schema is described below (Figure 3). Under basal conditions, CGI-58 localizes on the lipid droplet surfaces with perilipin 1, although ATGL exists predominantly within the cytoplasm [48], resulting in an attenuation of the interaction of ATGL with CGI-58 [49]. HSL is also located entirely in the cytoplasm, where it is nonphosphorylated and removed from lipid droplets, thereby reducing the hydrolysis activity of TG in adipocytes [36]. In contrast, hormonal activation of *β*-ARs-PKA provokes the association of CGI-58 with ATGL in fragmented lipid droplets following the rapid, within minutes, dissociation of PKA-phosphorylated perilipin 1 at Ser517 and CGI-58 [38, 48]. During that time, PKA promotes both phosphorylation and translocation of phosphorylated HSL at Ser659 and Ser660 from the cytoplasm to lipid droplets [50], and, in turn, perilipin 1 acts as a scaffold protein to bind HSL with lipid droplets [34], which results in an inducement of the maximal lipolytic response. Thus, serial modification events of lipolytic molecules, which support the localization of lipases, would play a critical role in the adaptive alteration of the lipolytic response in white adipocytes by physical exercise.

## 4. Effect of ET on the Number of ***β***-ARs, Which Is the First Step in the Mobilization of the Lipolytic Cascade

As mentioned in the above sections, stimulation of the *β*-ARs-AC system in white adipocytes results in a change in intracellular cAMP production and in the subsequent activation of PKA. Thus, an increase in the number of the *β*-ARs, which are expressed on the cell surfaces, would be expected to play a key role in the upregulation of lipolysis that is caused by ET. In ET, however, there is a small amount of evidence that indicates no change in the number of *β*-ARs [51, 52], which are measured by hydrophobic ligands, compared to the primary adipocytes of sedentary control rats. Moreover, investigation using hydrophilic ligands has demonstrated that the level of *β*-ARs on cell surfaces is significantly decreased due to ET in rat [53], indicating that the level of *β*-ARs by ET, at least in part, might be internalized into the cytoplasm rather than being increased on the cell surfaces. In addition, in rat, it has been shown that enhancement of *β*-ARs-AC coupling is observed in white adipocytes from ET [54, 55]. These results indicate that an ET-induced increase in lipolysis is not dependent on the number of *β*-ARs but rather the enhancement of the association efficiency of both *β*-ARs and Gs proteins. Thus, an ET-induced enhancement of lipolysis might be mediated by an adaptive alteration in post *β*-ARs.

**Figure 4:**
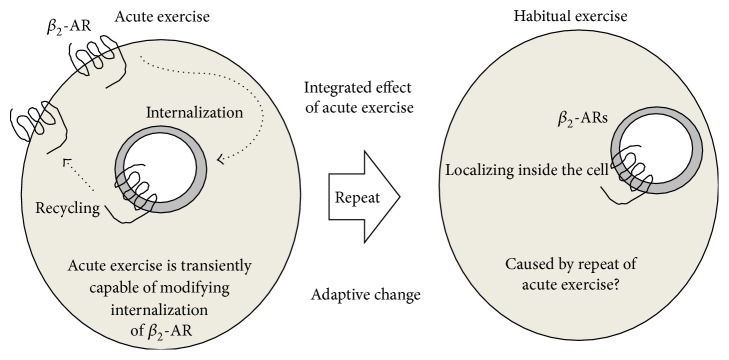
Internalization of *β*_2_-ARs from plasma membrane to intracellular space is modified by acute exercise in white adipocytes. HE-induced increase in the localizations of *β*-ARs into the cytoplasm might be a result of, at least in part, a mobilization of the trafficking of *β*_2_-ARs, which is caused by daily repeated bouts of acute exercise.

Under ET, repeat exposure of high levels of plasma catecholamines during bouts of daily exercise might be likely to trigger downregulation and change the localization of *β*-ARs into the cytoplasm in white adipocytes. Some very elegant studies conducted by Shenoy and coworkers [56] have shown that *β*_2_-ARs have a functional turnover cycle from the cellular surface to the cytosol via ubiquitination in a catecholamine dose-dependent manner. Of note, the adaptive change of adipocytes in response to ET appears to be the result of the integrative effect of bouts of acute exercise. Therefore, an understanding of the acute exercise-induced trafficking events of *β*_2_-AR would support the clarification of adaptive moderation of *β*-ARs by ET. In rat primary epididymal adipocytes, our results obtained from acute exercise demonstrated that localization of *β*_2_-ARs on the cell surface was upregulated at least 3 hours after exercise with reduced interaction of *β*-arrestin 2 and *β*_2_-AR, whereas it returned to the sedentary control levels 24 hours after exercise [57] (Figure 4). Loss of the combination of *β*-arrestin 2 and *β*_2_-AR resulted in a reduction in *β*_2_-AR ubiquitination, which thereby attenuated the internalization of *β*_2_-ARs into the cytoplasm. However, internalized *β*_2_-ARs were capable of quick recycling on the cell surface [58]. Together, the turnover of *β*_2_-ARs that was induced by every single bout of exercise might have been the result of the reduced levels of *β*_2_-ARs on the cell surfaces by ET, because, in this instance, there were no changes in the total amount of *β*-AR [51–53] (Figure 4).

## 5. Adaptive Alteration in G-Proteins by Habitual Physical Exercise

**Figure 5:**
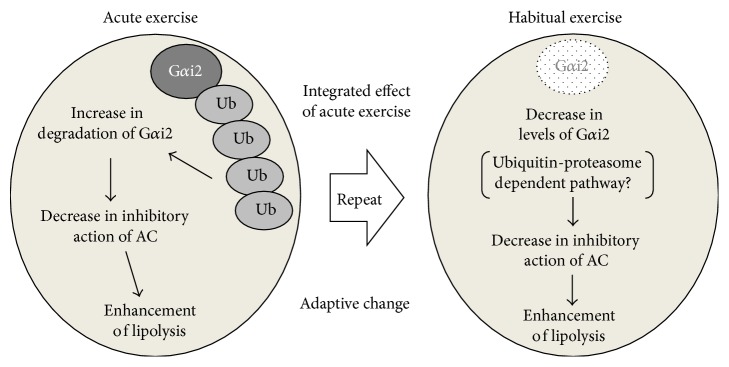
In white adipocytes, acute exercise accelerates the degradation of G*α*i2 proteins through the ubiquitin-proteasome system during, and at least 3 hours after, exercise. This mechanism might become a trigger for habitual decreases in the levels of G*α*i2 by ET.

It is known that both Gs protein *α* subunit (Gs*α*) and Gi protein *α* subunit (Gi*α*), which are dissociated from *β*- and *γ*-subunits by stimulation of *α*- and *β*-ARs, play key roles in the synergistic action of AC in white adipocytes. ET reportedly provoked a significant increase in AC activity of rat white adipocytes [59], accompanied by a decrease in the levels of Gi*α* protein, but caused no change in the levels of Gs*α* proteins in rat white adipocytes [60]. Moreover, ET significantly decreased the levels of Gi*α*2 protein, which predominantly inhibits AC activity, in rat white adipocytes [61] and in rat pancreatic islets [62]. These results indicate that ET positively regulates the signal transduction systems through the inhibition of Gi*α* function in adipose cells, which leads to the activation of AC. However, the mechanism(s) by which ET induces the downregulation of Gi*α*2 protein is unknown. In our previous study, acute exercise transiently downregulated the levels of Gi*α*2 proteins at least 3 hours after exercise via ubiquitin-proteasomal degradation machinery in rat white adipocytes [63] (Figure 5), suggesting the possibility that the downregulation of Gi*α*2 protein by ET might also be associated with acute exercise-induced proteolysis action, because ET is often defined by a repeat of bouts of acute exercise. Indeed, it is known that promotion of the ubiquitin-proteasome system is dependent on intracellular ATP, which is produced in several cells during exercise [9]. Moreover, the levels of MuRF-1, a muscle-specific E3 ligase, are reportedly reduced by ET in chronic heart failure patients [64]. Thus, in ET, the conspicuous effect of exercise on cellular energy production and selective transcriptional systems might be one of the triggers for the downregulation of Gi*α*2 proteins in white adipocytes (Figure 5). Such a conclusion, however, requires further study.

## 6. Manipulation of Lipolytic Molecules by Physical Exercise to Supply Energy

An understanding of the regulatory mechanisms underlying basal and hormone-stimulated lipolysis in adipocytes has evolved in recent years. However, little is known about the effect of ET on the molecular behavior of lipolytic proteins, that is, perilipin 1 and CGI-58, in white adipocytes. In rat, ET studies have shown no change in intracellular cAMP accumulation in white adipocytes compared with a sedentary control [51], suggesting the possibility that the molecular behavior of lipolytic proteins, which occur in the cell, plays a key role in the HE-induced enhancement of the lipolytic response. Indeed, our previous study indicated that white adipocytes obtained from ET rat enhance the levels of catalytic subunits of PKA proteins and PKA-anchoring protein 150 (AKAP150), which promotes the binding of PKA and its substrate, with activation of both PKA and HSL in the lipid droplet fraction of adipocyte homogenate [65]. These results would explain the phenomena whereby the ET-induced anchoring of AKAP150 to PKA enhances the magnitude of cAMP signaling in white adipocytes, even if accumulations of intracellular cAMP fail to increase as a result of ET. In rat, levels of HSL in adipocytes reportedly are upregulated by ET despite obesity [12] or normal circumstances in an individual [66], suggesting that the AKAP150-mediated enhancing action of PKA easily provokes the interaction of PKA with HSL, thereby activating the phosphorylation of HSL in cytoplasmic space. However, in rat white adipocytes, the phosphorylation of HSL by acute exercise is accompanied by an increase in intracellular cAMP production [63]. Thus, the functional alteration in AKAP150 might play a critical role in the adaptive augmentation of lipolytic responses by ET in white adipocytes.

**Figure 6:**
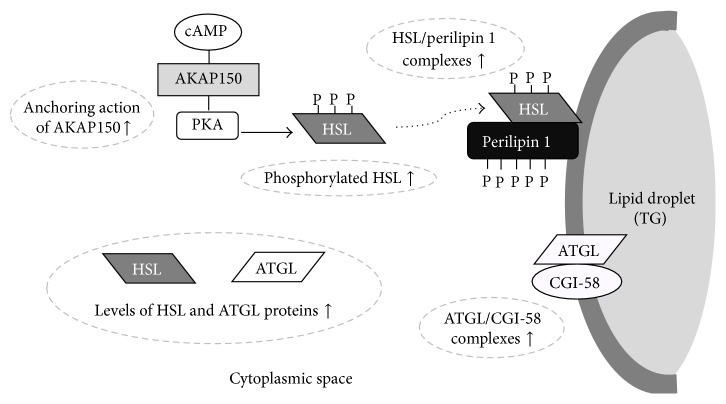
Summary of HE-induced adaptive changes of lipolytic molecules in white adipocytes. ET constantly promotes the expression levels of ATGL and HSL proteins. These conditions are likely to give rise to the stimulation of lipolytic responses through adaptive changes in molecules, such as increases in the anchoring action of AKAP150, higher levels of phosphorylated HSL, and augmentation of the formation of a complex of both HSL/perilipin 1 and ATGL/CGI-58 on the surface of lipid droplet. ↑: upregulation of function and expression levels of each molecule.

Alsted and colleagues were the first to report that levels of ATGL protein are significantly increased in human skeletal muscle by ET [67], although adipose tissue is used to identify ATGL [13]. It is noteworthy that the deletion of ATGL in mice impairs exercise performance [68] and that ATGL knockout mice show no increase in circulating FFA levels during exercise [69], suggesting that a molecular change in ATGL, as well as HSL, plays a role in supplying FFA from white adipocytes during physical exercise as a fuel for metabolism. To date, however, little is known about the effect of ET on the molecular changes of ATGL in white adipocytes. Recently, in rat, we demonstrated that mRNA, protein levels of ATGL, and HSL proteins all are upregulated by ET and that DNA-binding activities of peroxisome proliferation-activated receptor-*γ* 2 (PPAR-*γ*2) are closely associated with the ET-induced upregulation of ATGL [70]. Under these conditions, the binding of CGI-58 to ATGL was significantly increased on the lipid droplets with dissociations of CGI-58 and perilipin 1. These results indicate that the ET-induced acceleration of lipolytic responses is, at least in part, mediated by the hyperfunction of newly synthesized protein via the transcriptional activation of ATGL. Meanwhile, there is no evidence as to whether PKA-mediated phosphorylation of ATGL is involved in the hydrolysis of TG by ET, although at least one previous study has demonstrated that the increased phosphorylation of ATGL at Ser406, a PKA-mediated phosphorylation site, during both fasting and moderate single bouts of exercise is associated with an elevated rate of lipolysis in mice [71]. In our pilot study, ET showed higher levels of phosphorylated ATGL compared with the sedentary control in rat epididymal white adipocytes (unpublished data). These results suggest the possibility that ET might cause a phosphorylation-provoked conformational change in the protein structures of ATGL, which might result in a hypercombination of CGI-58 on lipid droplets [70], thereby enhancing the lipolytic responses in rat white adipocytes. In conclusion, several results have indicated that localization and/or phosphorylation of lipolytic molecules, such as perilipin 1, CGI-58, HSL, and ATGL, has a central function in the ET-induced adaptive alteration of lipolysis in white adipocytes and that the AKAP150-mediated activation of PKA also plays a key role in this mechanism (Figure 6).

## 7. Conclusion

It is well documented that exercise of moderate intensity accelerates the lipolytic responses in human white adipocytes [72–74]. In this review, studies showing both ET and acute exercise of light to moderate intensity [50–52, 56, 58–63, 65, 66, 70] indicated that moderate intensity of ET clearly provokes an enhancement of lipolysis in white adipocytes with an orchestral alteration in lipolytic molecules in a positive manner. However, little is known about the high-intensity exercise-induced behavior of lipolytic molecules in white adipocytes so far. Further studies are required to clarify this point.

A clarification of HE-induced molecular changes in a lipolytic cascade would apply not only to the prevention of obesity but also to the elucidation of a methodology for advances in exercise effectiveness. However, in white adipocytes no complete evidence exists to explain the mechanism(s) underlying the HE-induced adaptive changes in lipolysis. In particular, there are no new insights into the alterations in G protein-coupled receptors, nor into the family of G-proteins and related modification events brought about by HE, although a few results obtained in our studies have shown that ubiquitin-proteasome system plays a role in acute exercise-mediated amplification of the lipolytic cascade via the expression levels of both *β*_2_-AR and Gi*α*2 proteins. However, it is noteworthy that more than 200 genes that regulate lipid droplet morphology have been identified in* Drosophila* [75], suggesting that new molecules, which are unknown in mammalian species, would be related to the regulation of lipolytic events in white adipocytes with or without exercise. In the near future, the search for new molecules with the aim of elucidating their functions in an exercise-specific manner will shed new light on the calculations of a highly effective lipolytic system of exercise and will enhance the biological understanding of white adipocytes as a “vehicle” for the storage and supply of energy.

## Abbreviations

ET: Continuous exercise training
GPCRs: G-protein coupled receptors
-AR: Adrenergic receptor
-R: Receptor
Gs*α*: Gs protein *α* subunit
Gi*α*: Gi protein *α* subunit
AC: Adenylyl cyclase
P: Phosphorylation
PKA: cAMP-dependent protein kinase A
TG: Triacylglycerol
DG: Diacylglycerol
MG: Monoacylglycerol
IRS-1: Insulin receptor substrate-1
PI3-K: Phosphatidylinositol 3-kinase
PKB: Protein kinase B
PDE-3B: Phosphodiesterase-3B
HSL: Hormone-sensitive lipase
ATGL: Adipose triglyceride lipase
MGL: Monoacylglycerol lipase
AMPK: AMP activated protein kinase
CGI-58: Comparative gene identification-58
PAT/perilipin family proteins: Perilipin/perilipin 1, adipophilin/perilipin 2, TIP47/perilipin 3, S3-12/perilipin 4, and muscle lipid droplet protein/perilipin 5 family proteins
AKAP150: PKA-anchoring protein 150
PPAR-*γ*2: Peroxisome proliferation-activated receptor-*γ* 2.
